# Inhibition of Myeloperoxidase Activity in Cystic Fibrosis Sputum by Peptide Inhibitor of Complement C1 (PIC1)

**DOI:** 10.1371/journal.pone.0170203

**Published:** 2017-01-30

**Authors:** Pamela S. Hair, Laura A. Sass, Neel K. Krishna, Kenji M. Cunnion

**Affiliations:** 1 Department of Pediatrics, Eastern Virginia Medical School, Norfolk, Virginia, United States of America; 2 Children's Specialty Group, Norfolk, Virginia, United States of America; 3 Children’s Hospital of The King’s Daughters, Norfolk, Virginia, United States of America; 4 Department of Microbiology and Molecular Cell Biology, Eastern Virginia Medical School, Norfolk, Virginia, United States of America; Hospital for Sick Children, CANADA

## Abstract

Myeloperoxidase is the major peroxidase enzyme in neutrophil granules and implicated in contributing to inflammatory lung damage in cystic fibrosis. Free myeloperoxidase is present in cystic fibrosis lung fluid and generates hypochlorous acid. Here we report a new inhibitor of myeloperoxidase activity, Peptide Inhibitor of Complement C1 (PIC1). Using TMB as the oxidizing substrate, PIC1 inhibited myeloperoxidase activity in cystic fibrosis sputum soluble fractions by an average of a 3.4-fold decrease (P = 0.02). PIC1 also dose-dependently inhibited myeloperoxidase activity in a neutrophil lysate or purified myeloperoxidase by up to 28-fold (P < 0.001). PIC1 inhibited myeloperoxidase activity similarly, on a molar basis, as the specific myeloperoxidase inhibitor 4-Aminobenzoic acid hydrazide (ABAH) for various oxidizing substrates. PIC1 was able to protect the heme ring of myeloperoxidase from destruction by NaOCl, assayed by spectral analysis. PIC1 incubated with oxidized TMB reversed the oxidation state of TMB, as measured by absorbance at 450 nm, with a 20-fold reduction in oxidized TMB (P = 0.02). This result was consistent with an antioxidant mechanism for PIC1. In summary, PIC1 inhibits the peroxidase activity of myeloperoxidase in CF sputum likely via an antioxidant mechanism.

## Introduction

Myeloperoxidase (MPO) is a strong peroxidase present in neutrophil granules and its primary function is the generation of hypochlorous acid, the most powerful oxidant produced by neutrophils in appreciable amounts [1]. MPO catalyzes the production of hypochlorous acid in the presence of hydrogen peroxide and chloride anion [2]. MPO is present in the lung fluid of cystic fibrosis (CF) patients likely as the result of neutrophil degranulation or cell death [3, 4]. Multiple investigators have suggested that MPO in the lung fluid of CF patients may contribute to parenchymal destruction in addition to neutrophil elastase and other factors [5–7].

MPO consists of two light chains and two heavy chains plus a heme group that holds an iron atom [8] providing the peroxidase catalytic activity. The most commonly utilized substrate for testing MPO peroxidase activity is 3,3’,5,5’-Tetramethylbenzidine (TMB). Oxidation of TMB results in the loss of two hydrogen atoms, formation of TMB diimine [9] and a color change that can be read on a spectrophotometer. It has previously been shown that MPO incubation with H_2_O_2_ will generate hypochlorous acid that will subsequently oxidize and degrade the heme group causing release of the iron atom and loss of peroxidase activity [10]. The most common experimentally used inhibitor of MPO is 4-Aminobenzoic acid hydrazide (ABAH), which alters the charge state of the iron atom and irreversibly inactivates MPO in the presence of hydrogen peroxide by destruction of the heme ring [11].

Peptide Inhibitor of Complement C1 (PIC1) is a family of peptides 15 amino acids in length identified to inhibit the activation of C1 and the classical complement cascade [12, 13]. PIC1 peptides were originally derived from human Astrovirus 1 coat protein sequences[14, 15], but have subsequently undergone extensive rational drug design such that current derivatives demonstrate no significant homology with described proteins or peptides [16]. PIC1 binds C1q with nanomolar affinity similar to the cognate serine protease tetramer (C1r-C1s-C1s-C1r) and inhibits enzymatic activation [12]. The lead compound is PA-dPEG24 (IALILEPICCQERAA-dPEG24) [12]. We have previously shown that PIC1 (PA-dPEG24) can inhibit *P*. *aeruginosa*-initiated complement activation in CF sputum soluble fractions (sols) [17]. In attempting to further elucidate the ability of PIC1 to modulate complement-initiated neutrophil effectors, we identified a novel effect of PIC1 on MPO catalytic activity in CF sputum sols.

## Results

### PIC1 inhibition of MPO peroxidase activity in CF sputum sols

**Fig 1:**
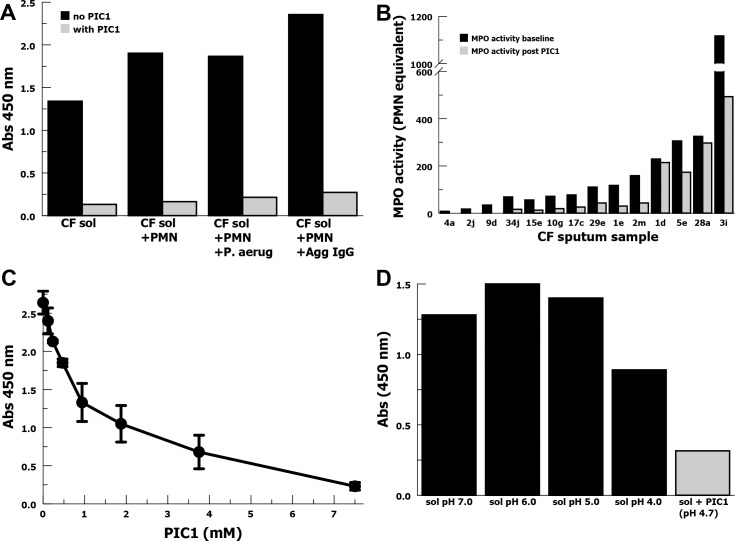
PIC1 inhibition of MPO peroxidase activity in CF sputum sol samples assayed by TMB. (A) MPO activity, ± PIC1, in a CF sputum sol at baseline and after addition neutrophils (PMN), killed *P*. *aeruginosa* (P. aerug) or heat-aggregated IgG (Agg IgG). (B) PIC1 (7.5 mM) inhibition of MPO activity in 14 CF sputum samples. (C) A dose-response titration of PIC1 inhibition of MPO activity for a CF sputum sol with moderate baseline MPO activity (n = 3). Data are means of independent experiments ±SEM. (D) MPO activity of CF sol in the presence of PIC1 (7.5 mM) or acidified with HCl.

In order to evaluate whether PIC1 could modulate complement-mediated neutrophil release of MPO, we tested a CF sputum sol with low baseline MPO activity. To this sol we added neutrophils (PMN) as well as degranulation stimulants either heat-killed *P*. *aeruginosa* or heat-aggregated IgG. Addition of PIC1 to the CF sol dramatically inhibited MPO oxidation of TMB in all conditions including CF sol only (**Fig 1A**). This suggested that PIC1 inhibited the peroxidase activity of MPO in the CF sputum sol; an unanticipated result indicating a complement-independent effect. Because MPO is believed to play a role in CF lung damage we then evaluated the effect of PIC1 across a broad range of baseline MPO activity in CF sputum sols. We selected 14 sputum samples from 12 CF patients representing a spectrum of MPO activity, assessed by TMB oxidation. With each sample we were able to show a decrease in MPO activity in the CF sputum sols in the presence of 7.5 mM PIC1 (**Fig 1B**). The median baseline MPO activity was 94.0 and after PIC1 median MPO activity decreased to 27.9 representing a 3.4-fold decrease (P = 0.02). In this figure MPO activity is represented as number of neutrophils lysed to yield an equivalent amount of TMB oxidation due to the wide range found for the sputum sols requiring the use of multiple dilution scales to make accurate measurements. We then performed a PIC1 dose-response experiment with a CF sputum sol with moderate MPO activity (**Fig 1C**). PIC1 yielded dose-dependent inhibition of MPO activity demonstrating an 11.5-fold reduction in MPO activity for 7.5 mM PIC1 compared with no PIC1 (P = 0.001). PIC1 is manufactured as an HCl salt and we questioned whether the oxidation of TMB in the assay could be affected by the increase in acidity. We tested MPO oxidation of TMB for a CF sol that was acidified with HCl to pH 4.0 (**Fig 1D**) and found minimal inhibition of MPO in contrast to PIC1 (pH 4.7). This suggested that PIC1 inhibition of MPO peroxidase activity in CF sol was not mediated by acidification of the sol.

### PIC1 inhibition of MPO peroxidase activity in neutrophil lysate or pure MPO

**Fig 2:**
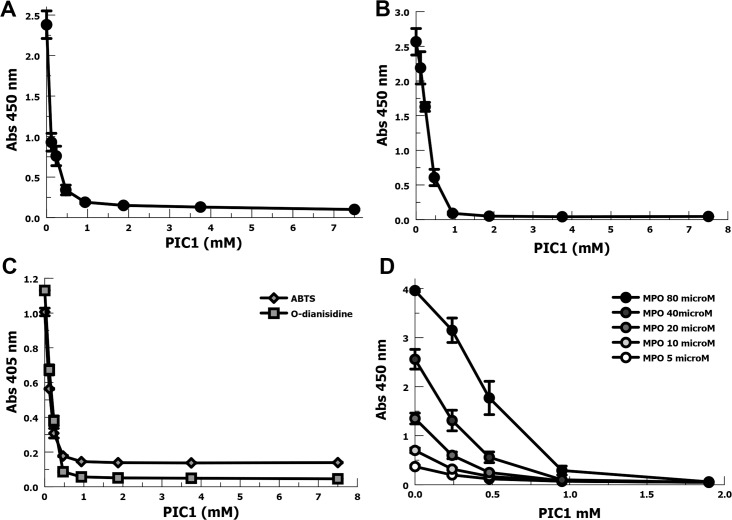
PIC1 inhibition of MPO peroxidase activity in neutrophil lysate or purified MPO. (A) Dose-response titration of PIC1 into a lysate of 10^6^ neutrophils assaying MPO activity with TMB (n = 4). Data are means of independent experiments ±SEM. (B) PIC1 dose-response inhibition of pure MPO (80 nM) oxidation of TMB (n = 3). Data are means of independent experiments ±SEM. (C) PIC1 inhibition of pure MPO oxidation of ABTS and O-dianisidine. (D) Dose-response inhibition of pure MPO at various concentrations by PIC1 (n = 3). Data are means of independent experiments ±SEM.

The predominant source of MPO in CF lung fluid is believed to come from neutrophils that have degranulated or died. In order to evaluate the effect of PIC1 on the peroxidase activity of freshly released MPO, we prepared neutrophil lysates by a detergent hypotonic lysis. We assayed dose-response with a titration of PIC1 into the neutrophil lysate and measured peroxidase activity with TMB (**Fig 2A**). Compared with no PIC1, 0.9 mM PIC1 inhibited MPO activity 12.5-fold (P < 0.001).

In order to directly test the ability of PIC1 to inhibit the peroxidase activity of MPO, we utilized purified MPO. PIC1 (0.9 mM) was able to inhibit purified MPO oxidation of TMB by 28-fold (P < 0.001) (**Fig 2B**). In order to test whether PIC1 inhibits MPO peroxidase activity for oxidizing multiple substrates and not merely TMB, we also tested ABTS (2,2'-azino-bis(3-ethylbenzothiazoline-6-sulphonic acid)) and O-dianisidine. Similar to results for TMB, PIC1 in the 0.5–0.9 mM range dramatically inhibited purified MPO oxidation of ABTS and O-dianisidine (**Fig 2C**). These results confirm that PIC1 inhibits MPO oxidation of multiple substrates. In order to evaluate PIC1 inhibition of MPO peroxidase activity over a range of purified MPO concentrations, we conducted more extensive dose-response experiments (**Fig 2D**). Previous studies have suggested that CF sputum sols will have MPO concentrations ranging from 0.5–20 μM [6, 18]. Concentration dependent PIC1 inhibition of MPO peroxidase activity was demonstrated for all MPO concentrations up to and including 80 μM. PIC1 at 0.5 mM inhibited MPO peroxidase activity 5.4-fold (P < 0.001) at the upper range of MPO in CF sputum, 20 μM.

### PIC1 comparison with ABAH

**Fig 3:**
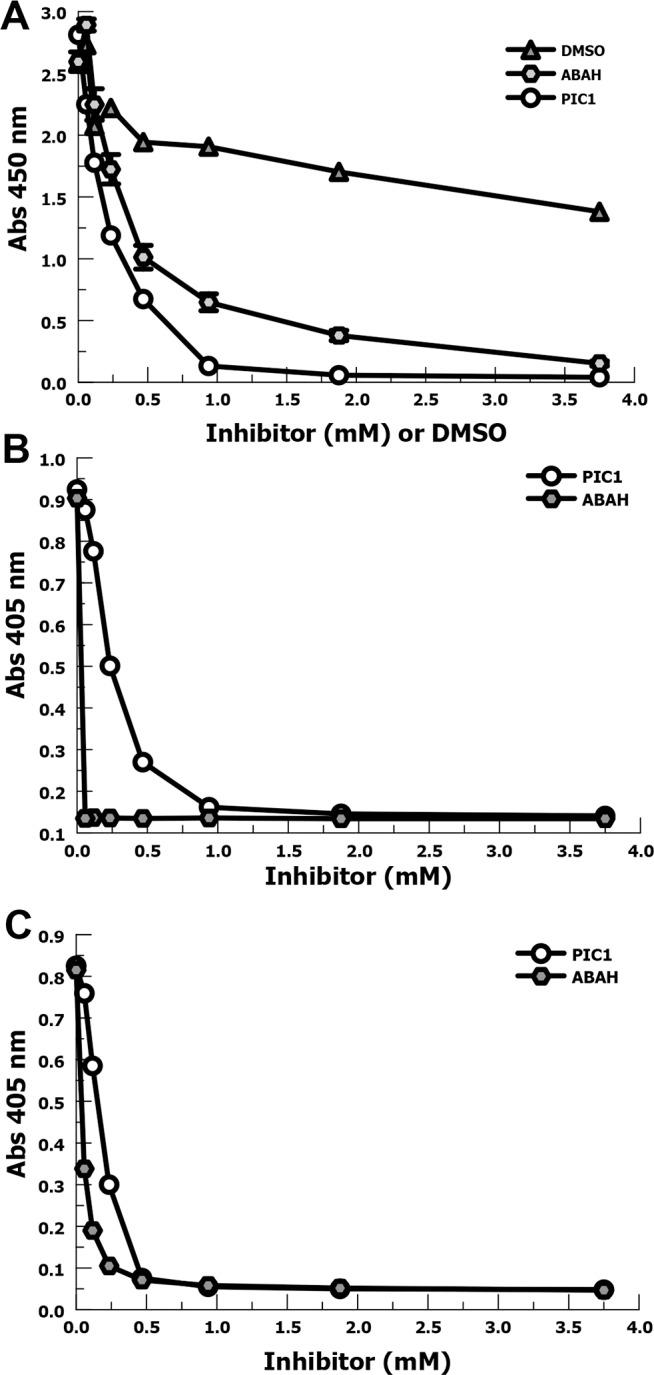
Comparison of PIC1 versus ABAH inhibition of purified MPO (80 nM) peroxidase activity. (A) Dose-response inhibition of MPO oxidation of TMB at various concentrations by PIC1, ABAH and DMSO (n = 3). Data are means of independent experiments ±SEM. (B) Comparison of PIC1 and ABAH inhibition of MPO oxidation of ABTS. (C) Comparison of PIC1 and ABAH inhibition of MPO oxidation of O-dianisidine.

We then compared PIC1 inhibition of purified MPO with a commonly utilized MPO inhibitor, 4-aminobenzoic acid hydrazide (ABAH). Because ABAH is initially solubilized in DMSO, we included a DMSO percent (v/v) matched control for each concentration of inhibitor tested. Our initial experiment utilized TMB as the oxidizing substrate (**Fig 3A**). DMSO alone appears to partially inhibit MPO activity suggesting that part of the inhibitory effect seen with ABAH is, at least in part, due to DMSO. PIC1 on a molar basis demonstrated similar or better inhibition of MPO oxidation of TMB compared with ABAH. At 0.9 mM PIC1 demonstrated a 7-fold (P = 0.002) improvement in MPO inhibition compared with ABAH. We then compared PIC1 versus ABAH inhibition of MPO oxidation of ABTS (**Fig 3B**) and O-dianisidine (**Fig 3C**). For these substrates PIC1 and ABAH showed similar inhibition of MPO peroxidase activity, but these substrates appeared to show somewhat greater sensitivity to ABAH inhibition of MPO peroxidase activity than was seen for TMB. In summary, these experiments demonstrate that PIC1 inhibits MPO peroxidase activity to a similar degree as ABAH, on a molar basis.

### Spectral analysis of the MPO heme ring

**Fig 4:**
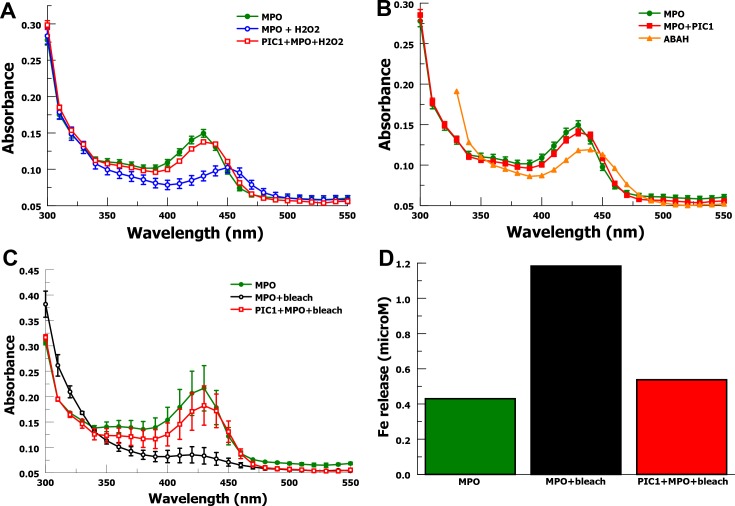
Spectral analysis of purified MPO heme ring. (A) MPO undergoes oxidative changes when incubated with H_2_O_2_. PIC1 protects MPO from oxidization in the presence of H_2_O_2_. Spectral absorption readings were taken from 300 to 550 nm (n = 3). Data are means of independent experiments ±SEM. (B) Spectral analysis of purified MPO when incubated with PIC1 or ABAH. Spectral absorption readings were taken from 300 to 550 nm (n = 3). Data are means of independent experiments ±SEM. (C) MPO undergoes oxidative changes in the presence of bleach. PIC1 protects MPO from oxidization in the presence of bleach. Spectral absorption readings were taken from 300 to 550 nm (n = 2). Data are means of independent experiments ±SEM. (D) Fe release from MPO in the presence of bleach is inhibited by PIC1 as measured by ferrozine assay.

PIC1 inhibition of MPO peroxidase activity could be due several mechanisms. PIC1 could alter the heme ring or the iron charge state preventing MPO peroxidase activity. PIC1 could be acting as an anti-oxidant protecting the target from oxidation. Previous investigators have shown that MPO interaction with hydrogen peroxide (H_2_O_2_) produces hypochlorous acid (HOCl) that oxidizes the heme ring of MPO and changes the charge state of the iron atom inactivating the enzyme [10]. Thus, MPO becomes a target of the peroxidase activity it generates. These changes to the heme ring and iron atom in MPO can be evaluated by measuring spectral absorption [10]. The spectral absorption for purified MPO is dramatically altered by exposure to H_2_O_2_ (**Fig 4A**). Hydrogen peroxide decreases MPO peak absorption at 430 nm (P = 0.0003), indicative of structural change to the heme ring, and shifts peak absorption to 450 nm, demonstrating a change in the charge state of the iron atom. When H_2_O_2_ is added to a mixture of MPO and PIC1 the wavelength of peak absorbance for MPO remains unchanged (430 nm) with an insignificant change in mean absorption (P = 0.10). This suggests that PIC1 is able to inhibit oxidative damage to the heme ring and alteration of the iron charge state. Since PIC1 appears to prevent structural change to MPO, it suggests that PIC1 inhibition of MPO peroxidase activity is via a mechanism other than by altering the MPO molecule.

MPO incubated with PIC1 does not significantly change the absorbance curve for MPO (**Fig 4B**), suggesting that PIC1does not alter the heme ring or the iron charge state of MPO. In contrast, MPO incubated with ABAH leads to a shift in wavelength peak to 440 nm due to a change in iron charge state, consistent with what has been previously reported [11]. These results suggest that PIC1 inhibits the peroxidase activity of MPO by a different mechanism than ABAH.

Bleach (NaOCl) produces similar oxidative spectral curve changes to MPO (**Fig 4C**) as has previously been reported for hypochlorous acid (HOCl) [10]. MPO in a mixture with PIC1 is largely protected from the oxidative effects of bleach, similar to our findings for H_2_O_2_. These findings suggest that PIC1 can inhibit the active peroxidase product of MPO. Previous investigators have shown that HOCl will cause iron release from hemoglobin resulting from oxidative damage to the heme ring [10]. We show similar results for MPO in the presence of bleach using a ferrozine assay (**Fig 4D**). PIC1 was able to prevent bleach-mediated release of iron from MPO consistent with protecting the heme ring from oxidative damage by bleach.

### Anti-oxidant effects of PIC1

**Fig 5:**
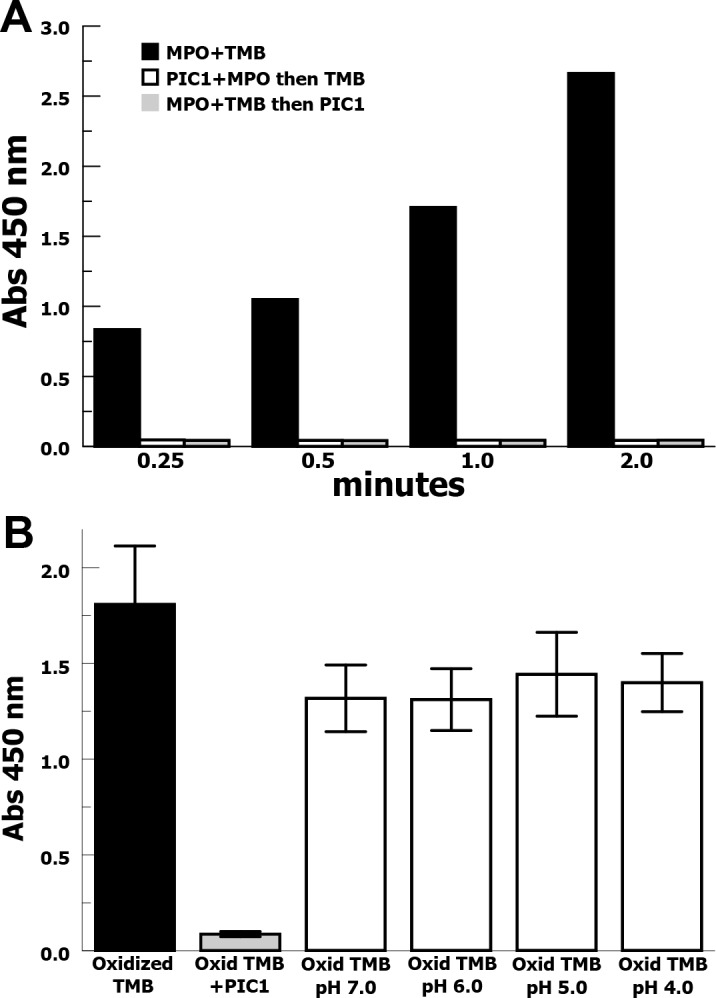
Analysis of reversal of TMB oxidation by PIC1. **(**A) Oxidation of TMB, as measured by absorbance, in the presence of MPO is inhibited when PIC1 is added before TMB or after TMB has been incubated with MPO. (B) MPO was bound to a well bottom and TMB was oxidized. The oxidized TMB solution was removed to an uncoated well and PIC1 was added or the solution was acidified with sulfuric acid and absorbance was measured (n = 4). Data are means of independent experiments ±SEM.

PIC1 inhibition of damage to the heme ring of MPO suggested that PIC1 may be inhibiting the peroxidase activity of MPO by an anti-oxidant effect. We then tested whether PIC1 could reverse the oxidized state of TMB after incubation with MPO. PIC1 was added to MPO prior to adding TMB as well as oxidizing TMB in the presence of MPO and then adding PIC1 (**Fig 5A**). When PIC1 was added after the MPO and TMB had incubated, the color change was reversed and the absorbance value was minimal, similar to adding TMB to a mixture of MPO and PIC1. This result shows that PIC1 can reverse the oxidation of TMB mediated by MPO. In order to prove that PIC1 was not mediating the reversal of TMB oxidation via MPO, we coated MPO on a well bottom and added TMB in solution to be oxidized. The oxidized TMB was then removed to an uncoated well, leaving the solid-phase MPO behind, and added PIC1 (**Fig 5B**). PIC1 reversed the oxidation state of TMB as measured by absorbance at 450 nm with a 20-fold reduction in oxidized TMB (P = 0.02). In order to demonstrate that this was not a pH effect, we oxidized TMB with solid-phase MPO, transferred the solution to a new well and acidified the solution with sulfuric acid. No reversal of TMB oxidation was seen at lower pH values demonstrating that pH was not the cause. Together, these results suggest that PIC1 has an anti-oxidant effect reducing oxidized TMB.

## Discussion

The peroxidase activity of MPO is typically contained intracellular most notably in neutrophil granules designed to fuse with phagocytized microorganisms to produce phagolysosomes. In this protected environment MPO can generate hypochlorous acid contributing to oxidative killing. However when MPO is released into the extracellular environment by neutrophil death or degranulation, it can mediate oxidative damage of host molecules including proteins, DNA and lipids leading to host tissue damage [19]. MPO-mediated inflammation has been implicated in multiple diseases including coronary artery disease [20, 21] and glomerular and tubulointerstitial kidney diseases [22]. Thus, the potential clinical utility of an MPO inhibitor amenable for human use appears to be high.

These experiments demonstrate that PIC1 can inhibit the peroxidase activity of purified MPO and MPO in CF sputum soluble fractions *in vitro*. Evidence from previous investigators implicate MPO as a major contributor to CF lung damage [5–7], which is consistent with MPO being the most prevalent peroxidase in neutrophils and the clear role of neutrophils mediating lung destruction in CF. Currently MPO is not a therapeutic target in CF treatment and the mostly commonly used MPO inhibitor *in vitro* is ABAH, which has poor aqueous solubility requiring dissolving in DMSO. DMSO as a diluent decreases the likelihood that ABAH be developed for human use due to the known toxicities of DMSO [23]. PIC1, in contrast, has excellent aqueous solubility and is well tolerated *in vivo* [12, 16, 24]. Our recent data suggests that dysregulated complement activation likely contributes to the inflammatory disease process in CF and PIC1 can inhibit *P*. *aeruginosa*-mediated generation of the pro-inflammatory anaphylatoxin C5a in CF sputum soluble fraction [17]. We speculate that PIC1 could potentially have two anti-inflammatory actions in CF lung fluid: 1) inhibit the generation of inflammatory complement effectors and 2) inhibit the peroxidase activity of MPO. These possibilities need to be explored using *in vivo* models.

Our data suggest that PIC1 inhibits the peroxidase activity of MPO via an anti-oxidant mechanism. PIC1 does not appear to directly affect the heme moiety of MPO, as assayed by spectral analysis. This is in contrast to ABAH, which changes the charge of the iron atom [11]. PIC1 appears to protect the heme-ring from oxidation mediated by H2O2 or NaOCl and prevents iron loss from the heme ring, which results from heme oxidation [10]. Additionally, PIC1 can reduce oxidized TMB, supporting an anti-oxidant mechanism for PIC1. The mechanism by which PIC1 mediates anti-oxidant activity is the subject of future studies.

## Materials and Methods

### Ethics statement

Sputum samples were collected as part of routine patient care during visits to the Children’s Hospital of The King’s Daughters Cystic Fibrosis Center. Written consent was obtained under an Eastern Virginia Medical School IRB approved protocol, 12-08-EX-0200. Blood from healthy donors was obtained with written consent under an Eastern Virginia Medical School IRB approved protocol, 02-06-EX 0216.

### Reagents

PIC1 (IALILEPICCQERAA-dPEG24) was manufactured by PolyPeptide Group (San Diego, CA) to ≥ 95% purity verified by HPLC and mass spectrometry analysis. Lyophilized PIC1 was solubilized in normal saline with 0.01 M Na_2_HPO_4_ buffer to 15 mM. Pure MPO (MPO) was purchased from Lee Biosolutions (Maryland Heights, MO). TMB as a solution containing H_2_O_2_ was purchased from Thermo Fisher Scientific (Waltham, MA). 2,2'-azino-bis(3-ethylbenzothiazoline-6-sulphonic acid) (ABTS) solution was purchased from Thermo Fisher Scientific. O-dianisidine and 4-aminobenzoic acid hydrazide (ABAH) were purchased from Sigma-Aldrich (St. Louis, MO).

### Sputum sols

Expectorated sputum samples were collected in sterile containers and immediately placed on ice [17]. The soluble fraction (sol) was extracted as a supernatant of free flowing liquid after cold (4°C) centrifugation at 14,000 g for 60 minutes, similar to previously described methods [25]. Due to the normal differences in CF sputum viscosity, sol fractions were not normalized for protein content consistent with previously described methods [26].

### Purified neutrophils and neutrophil lysate

Neutrophils from the blood of healthy volunteers were purified from heparinized blood by Hypaque-Ficoll step gradient centrifugation, dextran sedimentation, and hypotonic lysis, as previously described [27]. Neutrophils were adjusted to 1×10^7^ cells/ml for the functional studies. Neutrophils were sedimented, lysed in 1% Triton X-100, and 1×10^6^ cell/ml equivalent aliquots were used for neutrophil lysate assays.

### MPO peroxidase activity assays with CF sols

For CF sol and neutrophil experiments, 50 μl of CF sol was combined with 25 μl of buffer, 2.5×10^7^ heat killed *P*. *aeruginosa* (70°C for 15 min.), or heat-aggregated IgG (IVIg heated to 63°C for 20 min.) and incubated with or without 20 μl of PIC1 (at 15 mM) for 30 min. at 37°C. To these samples were added 1×10^6^ neutrophils for 1 hour at 37°C. These samples were then analyzed for MPO peroxidase activity by TMB assay, described below.

Assays of PIC1 inhibition of MPO peroxidase activity in clinical CF sols was performed by combining CF sols in a 1:1 ratio with 15 mM of PIC1 or PBS and incubating for 5 minutes before adding TMB. Additionally, one representative CF sol was incubated with increasing concentrations of PIC1, with a constant final volume, for 5 minutes and then adding TMB.

Assays of pH effect on MPO peroxidase activity in CF sols was performed by adjusting one representative CF sol, initially at a pH of 7.0, to pH values of 6.0, 5.0, and 4.0 using H_2_SO_4_. The same CF sol was also combined with an equal volume of PIC1 yielding a final pH of 4.7. TMB was added after 5 minutes.

### MPO peroxidase activity with neutrophil lysate or purified MPO

MPO peroxidase activity for neutrophil lysate was performed by combining the PMN lysate (equivalent to 1x10^6^ cell/ml) with increasing concentrations of PIC1, with a constant final volume, for 5 minutes before adding TMB. MPO peroxidase activity utilizing pure MPO was assayed by combing the MPO solution, diluted to 160 nM with PBS buffer, with an inhibitor, at denoted concentrations, in a 1:1 ratio (v/v) for 1 min. prior to adding TMB.

### Measurement of MPO peroxidase activity

To measure MPO peroxidase activity using TMB, 20 μl of assay supernatant, CF sol, or neutrophil lysate was serially diluted and then combined with 100 μl of TMB in a non-treated 96-well plate followed by 100 μl of 2.5 N H_2_SO_4_ (stop buffer) and absorbance was read at 450 nm [28]. For MPO functional assays using ABTS, 150 μl of ABTS solution was added to each sample and incubated for 5 minutes before adding 100 μl of 1% SDS to stop the reaction [29]. Absorbance was read at 405 nm. O-dianisidine working solution was prepared by making a 0.166 mg/ml solution in 50 mM phosphate-citrate buffer, pH 5.0 with a final of 0.006% H_2_O_2_ added immediately before use [30]. For MPO measurements, 100 μl of the prepared O-dianisidine solution was added to each sample and incubated for 2 minutes before adding 100 μl of 2.5 N H_2_SO_4_ to stop the reaction. Absorbance was read at 405 nm.

### Spectral analysis of MPO

Absorbance spectra experiments were performed in 0.1 ml PBS supplemented with 2 μM pure MPO followed by 2.5 mM PIC1, 2.5 mM ABAH, or 200 μM H_2_O_2_, or both, in a non-treated 96 well plate. As each component is added to the MPO it is allowed to incubate for 5 minutes. Pure MPO and bleach experiments were performed similarly using 2 μM MPO, 2.5 mM PIC1, or bleach (Clorox, Oakland, CA) at 1:1000 dilution, or both. Absorbance values were recorded from 300–550 nm [10].

### Ferrozine assay

Iron release from MPO was measured utilizing a ferrozine assay [10]. After the absorbance spectra was recorded, 100 μl of ascorbic acid (100 mM) was added to each sample and allowed to incubate for 5 minutes. Then 50 μl of ammonium acetate (16%) and 50 μl of ferrozine (16 mM) were added to the samples and mixed. After incubating for 5 minutes, absorbance was measured at 562 nm. A standard curve was prepared using ammonium Fe(III) sulfate and a linear regression was used to calculate the free iron concentration.

### PIC1 anti-oxidant assays with TMB

Pure MPO was coated onto an Immunlon-2 96 well plate at 24 μg/ml in carbonate buffer overnight at 4°C. The plate was washed to remove unbound protein with PBS and 0.05% Tween, and then 100 μl of TMB was added. Once the wells were fully developed, 100 μl of each sample was transferred to an uncoated coated well containing 20 μl of either water, PIC1, or ABAH, both at 7.5 mM. After 1 minute, 100 μl of 2.5 N H_2_SO_4_ was added to stop the reaction and absorbance was read at 450 nm.

### Statistical analysis

Quantitative data were analyzed determining means, standard error (SEM), and Student’s t-test [31] using Excel (Microsoft, Redmond, WA).

## Supporting Information

S1 Fig (XLSX)

S2 Fig (XLSX)

S3 Fig (XLSX)

S4 Fig (XLSX)

S5 Fig (XLSX)

## Abbreviations

CF: cystic fibrosis
MPO: myeloperoxidase
PIC1: Peptide Inhibitor of Complement C1
TMB: 3,3’,5,5’-Tetramethylbenzidine
ABAH: 4-Aminobenzoic acid hydrazide
PA-dPEG24: IALILEPICCQERAA-dPEG24
ABTS: 2,2'-azino-bis(3-ethylbenzothiazoline-6-sulphonic acid)
